# Transcriptome profiling of primary murine monocytes, lung macrophages and lung dendritic cells reveals a distinct expression of genes involved in cell trafficking

**DOI:** 10.1186/1465-9921-10-2

**Published:** 2009-01-16

**Authors:** Zbigniew Zasłona, Jochen Wilhelm, Lidija Cakarova, Leigh M Marsh, Werner Seeger, Jürgen Lohmeyer, Werner von Wulffen

**Affiliations:** 1 Department of Internal Medicine, Division of Pulmonary and Critical Care Medicine, University of Giessen Lung Center, Klinikstr. 36, 35392 Giessen, Germany; 2 Institute for Pathology, University of Giessen Lung Center, Langhansstr 10, 35392 Giessen, Germany

## Abstract

**Background:**

Peripheral blood monocytes (PBMo) originate from the bone marrow, circulate in the blood and emigrate into various organs where they differentiate into tissue resident cellular phenotypes of the mononuclear phagocyte system, including macrophages (Mϕ) and dendritic cells (DC). Like in other organs, this emigration and differentiation process is essential to replenish the mononuclear phagocyte pool in the lung under both inflammatory and non-inflammatory steady-state conditions. While many studies have addressed inflammation-driven monocyte trafficking to the lung, the emigration and pulmonary differentiation of PBMo under non-inflammatory conditions is much less understood.

**Methods:**

In order to assess the transcriptional profile of circulating and lung resident mononuclear phagocyte phenotypes, PBMo, lung Mϕ and lung DC from naïve mice were flow-sorted to high purity, and their gene expression was compared by DNA microarrays on a genome-wide scale. Differential regulation of selected genes was validated by quantitative PCR and on protein level by flow cytometry.

**Results:**

Differentially-expressed genes related to cell traffic were selected and grouped into the clusters (i) matrix metallopeptidases, (ii) chemokines/chemokine receptors, and (iii) integrins. Expression profiles of clustered genes were further assessed at the mRNA and protein levels in subsets of circulating PBMo (GR1- vs GR1+) and lung resident macrophages (alveolar vs interstitial Mϕ). Our data identify differentially activated genetic programs in circulating monocytes and their lung descendents. Lung DC activate an extremely diverse set of gene families but largely preserve a mobile cell profile with high expression levels of integrin and chemokine/chemokine receptors. In contrast, interstitial and even more pronounced alveolar Mϕ, stepwise downregulate gene expression of these traffic relevant communication molecules, but strongly upregulate a distinct set of matrix metallopetidases potentially involved in tissue invasion and remodeling.

**Conclusion:**

Our data provide new insight in the changes of the genetic profiles of PBMo and their lung descendents, namely DC and Mϕ under non-inflammatory, steady-state conditions. These findings will help to better understand the complex relations within the mononuclear phagocyte pool of the lung.

## Background

Peripheral blood monocytes (PBMo) can emigrate from the blood through the endothelial barrier into various tissues under both non-inflammatory, steady-state conditions and in response to inflammatory stimuli. After extravasation, PBMo undergo rapid phenotype changes and differentiate into cells of the organ resident mononuclear phagocyte system, namely macrophages (Mϕ) and dendritic cells (DC) [1,2]. This highly coordinated process implicates close linkage between monocyte trafficking and cellular differentiation, which shapes the phenotype of the extravasated cells. Monocyte differentiation has been extensively studied *in vitro*. Monocytes cultured in medium containing macrophage colony-stimulating factor (M-CSF) differentiate into Mϕ, while in the presence of granulocyte macrophage colony-stimulating factor (GM-CSF) and Interleukin (IL) -4, monocytes differentiate into DC [3,4]. Although recent *in vivo *investigations have demonstrated that subsets of PBMo can be precursors for DC and Mϕ [5,6], the detailed fate of PBMo once they leave the circulation has not been comprehensively addressed. Moreover, while cell recruitment under inflammatory conditions has been extensively studied, the tissue migration and differentiation of mononuclear phagocytes under non-inflammatory conditions remain poorly understood.

In the lung, cells of the mononuclear phagocyte system are key players in host defense and immunological homeostasis. While Mϕ are generally present in both the lung interstitium and alveolar airspaces, DC are mainly located within the interstitium with only a minor proportion found at the respiratory tract surface areas [7,8]. In addition to their different localization, Mϕ and DC in the lung fulfill distinct and specialized roles in the immune response, which correlate with their different migration properties and cellular phenotypes. In the absence of inflammatory stimuli, DC have a much shorter half-life in the lung compared to Mϕ [9]. Furthermore, DC do not exhibit impressive phagocytic activity, but rather process antigens which are then presented to T cells upon stimulation, causing antigen specific T cell priming. To ensure an effective antigen presentation to T cells, DC must migrate to the regional lymph nodes. In contrast, Mϕ are considered to form resident cell populations both in the interstitium (interstitial macrophages, iMϕ) and in the alveolar airspace (resident alveolar macrophages, rAM), where they function as major sentinel and phagocytic population of the lung for invading pathogens [10]. Alveolar macrophage and DC precursors must migrate from the bloodstream through endothelial and epithelial barriers into the alveolar compartment. This journey requires the expression of genes involved in communication with barrier structures and rapid adjustment to different oxygen concentrations and osmotic pressures.

Trafficking of monocytes into lung tissue and their differentiation into lung resident Mϕ and DC is supposed to be regulated by the expression of specific gene clusters, which promote cell-cell interaction, migration and matrix degradation and the acquisition of tissue specific cellular phenotypes. Traffic related gene clusters include chemokines, integrins, and tissue-degrading matrix metallopeptidases (Mmps), for all of which members have been shown to be functionally important. A complete picture, however, of the gene clusters that are regulated during *in vivo *migration and differentiation of PBMo under non-inflammatory conditions has not yet been obtained. Currently, adaptive changes of cellular phenotypes cannot be directly assessed by cell fate mapping during the slow trafficking of mononuclear phagocytes to lung tissue under steady-state conditions. Therefore, as an alternative approach to gain a better insight into the genetic programs that drive the mononuclear phagocyte migration and differentiation processes, the transcriptomes of circulating monocytes were compared with their lung tissue mononuclear phagocyte progeny. By this approach, gene clusters related to cell migration were identified and confirmed by quantitative real-time PCR (qRT-PCR) analysis that are differentially expressed between PBMo versus lung Mϕ and DC, and which shape the mononuclear phagocyte phenotyes in the circulation and in the lung tissue.

## Methods

### Mice

Experiments were performed with wild-type C57BL/6N mice (six to nine weeks old), which were purchased from Charles River (Sulzbach, Germany) and were maintained under specific pathogen free conditions with free access to food and water. All animal experiments were approved in accordance with the guidelines of Institutional Animal Care and Use Committee and were approved by the local government authority.

### Isolation of peripheral blood monocytes, lung macrophages and lung DC

Mice were sacrificed by an overdose of isoflurane inhalation (Forene^®^, Abbott). Blood was collected from the *vena cava caudalis *and aseptically transferred to 15 ml tubes. Clotting was prevented by addition of EDTA. Erythrolysis was performed with 10 ml 0.8% ammonium chloride lysis buffer. Erythrolysis was stopped by addition of 5 ml of RPMI-1640 medium supplemented with 10% FCS and L-glutamine, and cells were centrifuged (400 × *g*, 10 min, 4°C). The pellet was re-suspended in 10 ml ammonium chloride buffer, and the procedure was repeated. Cells were then washed in 5 ml RPMI-1640 medium supplemented with 10% FCS and L-glutamine, resuspended in PBS/2 mM EDTA/0.5% FCS, and stained for flow cytometry as outlined below.

Macrophages and DC from lungs were isolated as described in detail recently [8,11]. Briefly, lungs were perfused with 20 ml of sterile HBSS until free of blood by visual inspection, then removed and transferred into Petri dishes containing 0.7 mg/ml collagenase A (Roche;) and 50 μg/ml DNAse I (Serva;) in RPMI-1640 medium. Lungs were minced and cut into small pieces, agitated on a shaker (30 min, RT) and then incubated at 37°C for 30 min in a humidified atmosphere containing 5% CO_2_. Cell aggregates were dispersed by repeated passage through a syringe, and filtered through a 200 μm and a 40 μm cell strainer (BD Biosciences), to obtain single cell suspension. Subsequently, cells were rinsed with HBSS and PBS/2 mM EDTA/0.5% FCS, followed by incubation with an excess concentration of unspecific IgG (Octagam, Octapharma, Germany) to reduce non-specific antibody binding. After washing with PBS/2 mM EDTA/0.5% FCS, cells were stained with magnetic bead-conjugated anti-CD11c antibodies (Miltenyi Biotec) followed by magnetic separation according to the manufacturer's instructions. Subsequently, the cell population (containing CD11c positive cells) was stained with CD11c-PE conjugated antibodies (BD Pharmingen) and sorted as outlined below.

To obtain resident alveolar macrophages, bronchoalveolar lavage (BAL) was performed with 500 μl aliquots of sterile PBS/2 mM EDTA (pH 7.2) until a BAL fluid (BALF) volume of 5 ml was recovered following previously described protocols [12]. The BALF was centrifuged (400 × g, 10 min, 4°C); the cell pellet was resuspended in PBS/2 mM EDTA/0.5% FCS, stained with CD11c PE conjugated antibodies (BD Pharmingen) and subjected to sorting.

### Flow cytometric analysis and flow sorting

For staining for flow cytometric analysis and sorting, cells were resuspended in PBS/2 mM EDTA/0.5% FCS. Cell numbers were assessed using a Neubauer chamber. Fc-receptor-mediated and non-specific antibody binding was blocked by addition of excess non-specific immunoglobulin (Octagam^®^, Octapharma, Germany). The following monoclonal antibodies were used at appropriate dilutions for staining: CD11c-PE and -APC (HL3, BD Pharmingen), CD11b-FITC, -APC, and -PE (M1/70, BD Pharmingen), CD115-PE (604 B5 2EII, Serotec), GR-1-PE-Cy7 and -PE (RB6-8C5, Biolegend), F4/80-PE (CI:A3-1, Serotec), biotinylated I-A/I-E (2G9, BD Pharmingen), CD3-PE (17A2, BD Pharmingen), CD19-PE (1D3, BD Pharmingen), NK-1.1-PE (PK136, BD Pharmingen), CD80-PE (1G10, BD Pharmingen), CD86-PE (GL1, BD Pharmingen), B220-PE (RA3-6B2, BD Pharmingen), CD49d-PE (R1-2, Biolegend), CD103-PE (2E7, Biolegend), CD61-PE (2C9G2, Biolegend), Integrin β7 (FIB504, Biolegend).

Staining was performed at 4°C in the dark for 20 min. After staining, cells were washed twice in PBS/2 mM EDTA/0.5% FCS. Biotinylated primary antibodies were further incubated for 5 min with APC-conjugated streptavidin (BD Pharmingen), followed by two additional washes with PBS/2 mM EDTA/0.5% FCS. Cell sorting was performed with a FACSVantage SE flow cytometer equipped with a DiVA sort option and an argon-ion laser at 488 nm excitation wavelength and a laser output of 200 mW (BD Biosciences). A FACSCanto flow cytometer (BD Biosciences) was used for flow cytometric characterization of cell populations. The BD FACSDiVa software package was used for data analysis (BD Biosciences). Purity of sorted cells was ≥ 98% as determined by flow cytometry and differential cell counts of Pappenheim (May-Grünwald-Giemsa)-stained cytospins.

### RNA isolation and cDNA synthesis

After sorting, cells were frozen at -80°C in RLT lysis buffer (Qiagen) with 1% β-mercaptoethanol (Sigma). RNA from highly purified cell populations was isolated using an RNeasy Micro Kit (Qiagen) according to the manufacturer's instructions. Quantification and purity of RNA was determined with an Agilent Bioanalyzer 2100 (Agilent Biosystems). Only those RNA preparations exceeding absorbance ratios of A_260/280*nm *_> 1.90 and of a total amount of RNA greater than 200 ng were used for microarray experiments. The cDNA synthesis, reagents and incubation steps were performed as described previously [13].

### Microarray experiments

A total of 32 animals were used for the microarray experiments. From one mouse, three different cell types, namely PBMo, Mϕ, and DC, were sorted as outlined above, and RNA was extracted. In order to get enough RNA for a labeling reaction, RNA was pooled from 4 different extractions (4 mice, one pool). Two pools of labeled amplified RNA (aRNA) from different cell types were used per microarray hybridization (one per dye to reach a balanced dye swap, see below). The total number of 12 hybridizations were performed with each 4 hybridizations comparing PBMo with Mϕ, PBMo with DC, and Mϕ with DC, respectively.

The sample preparation (reverse transcription, T7 RNA amplification, labeling, purification, hybridization and subsequent washing and drying of the slides) was performed according to the Two-Color Microarray-Based Gene Expression Analysis Protocol version 5.5 using the Agilent Low RNA Input Linear Amplification Kit (Agilent Technologies, Wilmington, DE). Per reaction, 1 μg of total RNA was used. The samples were labeled with either Cy3 or Cy5 to match a balanced dye-swap design. The Cy3- and Cy5-labeled RNA pools were hybridized overnight to 4 × 44 K 60 mer oligonucleotide spotted microarray slides (Mouse Whole Genome 4 × 44 K; Agilent Technologies). The dried slides were scanned using a GenePix 4100A Scanner (Axon Instruments, Downingtown, PA). Image analysis was performed with GenePix Pro 5.0 software. Data were evaluated using the R software [14] and the limma package [15] from BioConductor [16]. The spots were weighted for subsequent analyses according to the spot intensity, homogeneity, and saturation. The spot intensities were corrected for local background using the method of Edwards [17] with an offset of 64 to stabilize the variance of low-intensity spots. The M/A data were LOESS normalized [18] before averaging. Genes were ranked for differential expression using a moderated t-statistic [19]. Statistics were obtained by extracting the contrasts of interest after fitting an overall model to the entire dataset. Candidate lists were created by selecting genes with more than a two-fold difference in expression by keeping a false-discovery rate of 10%. The adjustment for multiple testing was done with the method of Benjamini and Hochberg [20]. Pathway analyses were performed using Pathway-Express from Onto-Tools [21]. The complete data set is accessible online in the GEO database  under the accession number GSE13558.

### Validation of genes by quantitative real time RT-PCR

To validate the results obtained by microarray, RNA transcripts of selected genes were analyzed on independently sorted samples by qRT-PCR using the ΔC_T _method for the calculation of relative changes [22]. The *beta-actin and gapDH *genes were confirmed by qRT-PCR to be ubiquitously and consistently expressed genes among the different cell types analyzed in this study (data not shown), and their averaged expression was used as reference gene. The qRT-PCR analysis was performed with a Sequence Detection System 7900 (PE Applied Biosystems). Reactions (final volume: 25 μl) were set up with the SYBR™Green PCR Core Reagents (Invitrogen), 5 μl cDNA sample and 45 pmol forward (f) and reverse (r) primers. The intron-spanning primer sequences used were: Itgam, 5'-GGA CTC TCA TGC CTC CTT TG-3' (f), 5'-ACT TGG TTT TGT GGG TCC TG-3' (r); Itgb3, 5'-GTC CGC TAC AAA GGG GAG AT-3' (f), 5'-TAG CCA GTC CAG TCC GAG TC-3' (r); Itgb7, 5'-GAG GAC TCC AGC AAT GTG GT-3' (f), 5'-GGG AGT GGA GAG TGC TCA AG-3' (r); Itga4, 5'-TTC GGA AAA ATG GAA AGT GG-3' (f), 5'-AAC TTT TGG GTG TGG CTC TG-3' (r); Itgae, 5'-TGG CTC TCA ATT ATC CCA GAA-3' (f), 5'-CAT GAC CAG GAC AGA AGC AA-3' (r); Adamts2, 5'-AGT GGG CCC TGA AGA AGT G-3' (f), 5'-CAG AAG GCT CGG TGT ACC AT-3' (r); Adam19, 5'-GCT GGT CTC CAC CTT TCT GT-3' (f), 5'-CAG AAC TGC CAA CAC GAA GA-3' (r); Adam23, 5'-GCT CCA CGT ATC GGT CAA CT-3' (f), 5'-CCC ACG TCT GTA TCA TCG TCT-3' (r); Mmp12, 5'-TGA TGC AGC TGT CTT TGA CC-3' (f), 5'-GTG GAA ATC AGC TTG GGG TA-3' (r); Mmp13, 5'-ATC CCT TGA TGC CAT TAC CA-3' (f), 5'-AAG AGC TCA GCC TCA ACC TG-3' (r); Mmp14, 5'-GCC CAA TGG GAA GAC CTA CT-3' (f), 5'-AGG GTA CTC GCT GTC CAC TG-3' (r); Mmp19, 5'-TCC AGT GAC TGC AAA ACC TG-3' (f), 5'-AGT CGC CCT TGA AAG CAT AA-3' (r); Ccl2, 5'-AGC ATC CAC GTG TTG GCT C-3' (f), 5'-CCA GCC TAC TCA TTG GGA TCA T-3' (r); Ccr2, 5'-TCT TTG GTT TTG TGG GCA ACA-3' (f), 5'-TCA GAG ATG GCC AAG TTG AGC-3' (r); Ccl5, 5'-CTG CTT TGC CTA GGT CTC CCT-3' (f), 5'-CGG TTC CTT CGA GTG ACA AAC-3' (r); Ccr7, 5'-GTG GTG GCT CTC CTT GTC AT-3' (f), 5'-GAA GCA CAC CGA CTC GTA CA-3' (r); IL-18, 5'-CTG GCT GTG ACC CTC TCT GT-3' (f), 5'-CTG GAA CAC GTT TCT GAA AGA AT-3' (r); beta-actin, 5'-ACC CTA AGG CCA ACC GTG A-3' (f), 5'-CAG AGG CATA CAG GGA CAG CA-3' (r); GapDH, 5'-TGG TGA AGG TCG GTG TGA AC-3' (f), 5'-TGA ATT TGC CGT GAG TGG AG-3' (r). Data analysis and statistics were performed using the R program. All data are displayed as mean values ± SD. Statistical differences between treatment groups were estimated by ANOVA with Turkey's *post hoc *test for multiple comparisons. Differences were considered statistically significant when *p *values were < 0.05.

## Results

### Immunophenotypic identification and high purity isolation of PBMo, lung DC and lung Mϕ used for transcriptome profiling

**Figure 1:**
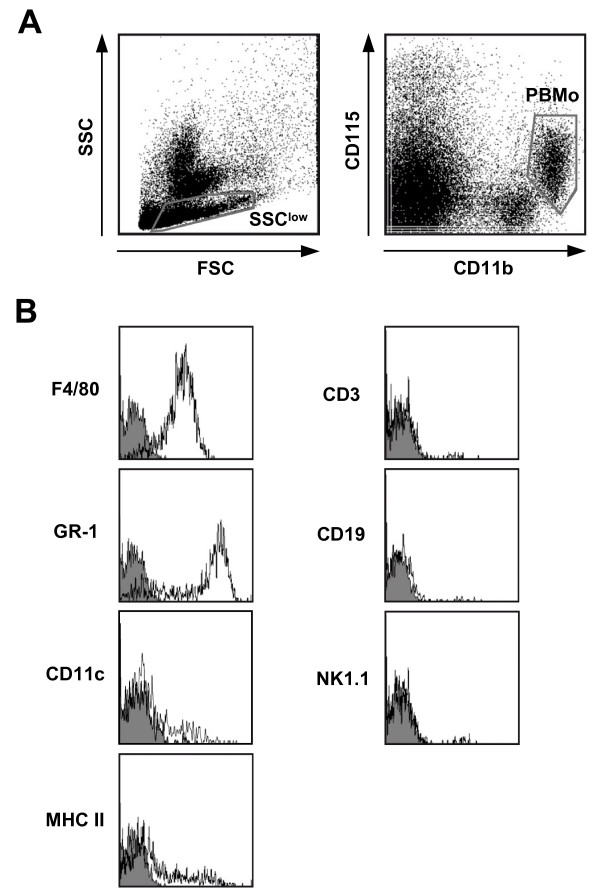
**Identification and characterization of PBMo by flow cytometry**. **A) **Peripheral blood was obtained from untreated mice as described, subjected to erythrolysis, and analyzed by flow cytometry. PBMo were identified as low side scatter (SSC) cell population showing a cell surface expression of CD11b and CD115. **B) **The cell surface antigen distribution profile of PBMo was characterized by flow cytometry. PBMo were gated as displayed in (**A**). Open histograms indicate specific fluorescence of the respective antigen; shaded histograms represent control stained cells. Note that all cells displayed F4/80 expression, but were negative for GR-1, CD3, CD19, B220/CD45R, and NK1.1, thus excluding contamination by neutrophils, T cells, B cells, or NK cells, respectively. Displayed data are representative of three independent experiments.

For high purity sorting, PBMo were identified as SSC^low^, CD11b^pos^, M-CSF receptor/CD115^pos ^cells following previously reported protocols [23] (Fig. 1A). The cells defined by this approach homogenously expressed the monocyte marker F4/80, and were partially positive for GR-1 and CD11c, with low levels or absence of MHC class II expression, thereby exhibiting the typical phenotype of PBMo [23]. In contrast, no expression of T cell, B cell or NK cell markers (CD3, CD19, and NK1.1, respectively) was detected (Fig. 1B).

**Figure 2:**
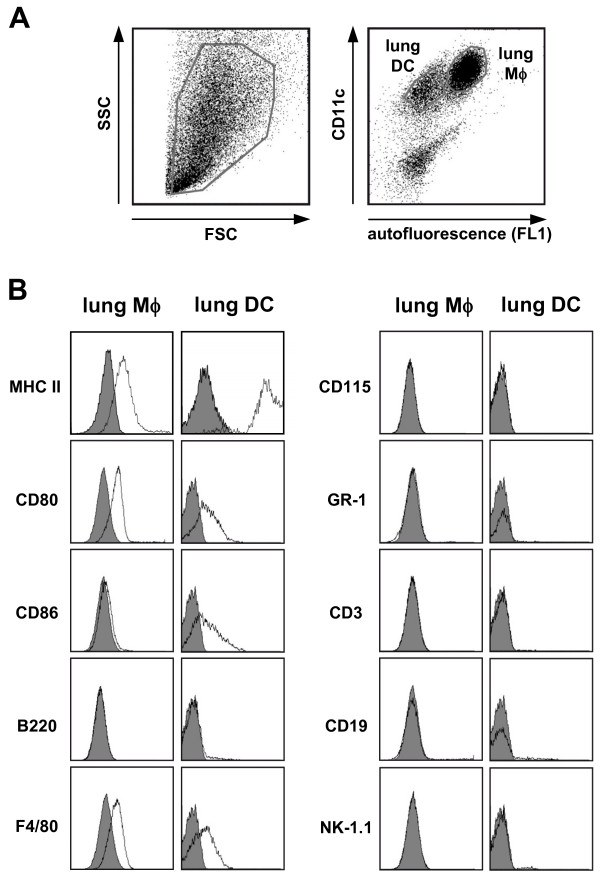
**Identification and characterization of lung Mϕ and DC by flow cytometry**. **A) **CD11c positive cells were obtained from lung homogenate by magnetic bead isolation, stained for CD11c, and analyzed by flow cytometry. Lung DC and lung Mϕ were differentiated by CD11c expression and autofluorescence with lung DC displaying a low autofluorescence and lung Mϕ displaying a high autofluorescence in the FL1 channel. **B) **The cell surface antigen distribution profiles of lung Mϕ and lung DC were analyzed by flow cytometric analysis. Lung Mϕ and DC were gated as displayed in (**A**). Open histograms indicate specific fluorescence of the respective antigen; shaded histograms represent control stained cells. Displayed data are representative of three independent experiments.

For high purity separation of Mϕ and DC from lung homogenates, in a first step the CD11c^pos ^cell fraction was isolated from lung homogenates using magnetic bead separation as outlined in the Materials and Methods section. Within this cell population, lung DC were identified as CD11c^pos^, low autofluorescent cells in the FL1 channel, while lung Mϕ were discriminated as CD11c^pos^, high FL1 autofluorescent cells (Fig. 2A). Further phenotyping of accordingly gated subsets revealed the characteristic marker profiles of lung DC and Mϕ, with lung DC displaying a MHC II^high ^CD80^low ^CD86^low ^F4/80^low ^phenotype and lung Mϕ displaying a MHC II^low ^CD80^low ^CD86^neg ^F4/80^pos ^phenotype (Fig. 2B), which were in line with previously published results [8,11]. Lung DC primarily exhibited an immature phenotype, as defined by high expression of MHCII and intermediate expression of the co-stimulatory molecules CD80 and CD86 (Fig. 2B). Neither an expression of CD115 nor of neutrophil, T cell, B cell, or NK cell markers was detected (Fig. 2B). The purity of sorted cells used for the microarray experiments (PBMo, lung DC and lung Mϕ) was assessed by flow cytometry and Pappenheim-stained cytospins and was always ≥ 98%. As sample processing may alter the gene expression profile of primary cells [24], every effort was made to minimize processing time and, where possible, all procedures were performed on ice.

### Differentially expressed genes between PBMo, lung DC and lung Mϕ

After cell sorting and RNA isolation, gene expression profiles of PBMo, lung DC and lung Mϕ were compared by DNA microarray on a whole genome scale. For each comparison, four hybridizations were performed. Genes that exhibited a greater than two-fold change in expression were considered as being differentially expressed, as described in the Materials and Methods section. Among the genes differentially expressed between lung Mϕ and PBMo, 1530 genes were up-regulated, and 1440 genes were down-regulated. Comparing lung DC and PBMo, 1271 genes were up-regulated, and 341 were down-regulated. Furthermore, 832 genes were found to be up-regulated and 1565 genes down-regulated between lung Mϕ and DC. An analysis of the correlation of the M values for the regulated genes from the different hybridizations showed a high correlation with an average Pearson correlation coefficient of 0.95, indicating a high consistency between the four hybridizations per group. In a pathway analysis, using *Pathway-Express from Onto-Tools*, the cell adhesion molecule pathway was the most differentially regulated pathway in all comparisons. The antigen presentation and processing pathway was the second most differentially regulated pathway comparing lung Mϕ versus DC and DC versus PBMo.

**Figure 3:**
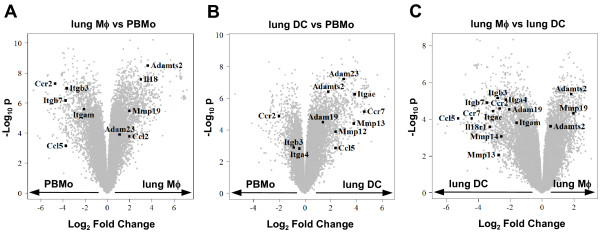
**Volcano plot representation of microarray data**. Gene expression profiles of **A) **lung Mϕ versus PBMo, **B) **lung DC versus PBMo, and **C) **lung Mϕ versus lung DC were plotted according to the log_2 _fold change (X axis) and log_10_unadjusted p-value (Y axis). The genes for which the expression has been validated by qRT-PCR are highlighted. Data are representative of four hybridizations per group.

**Figure 4:**
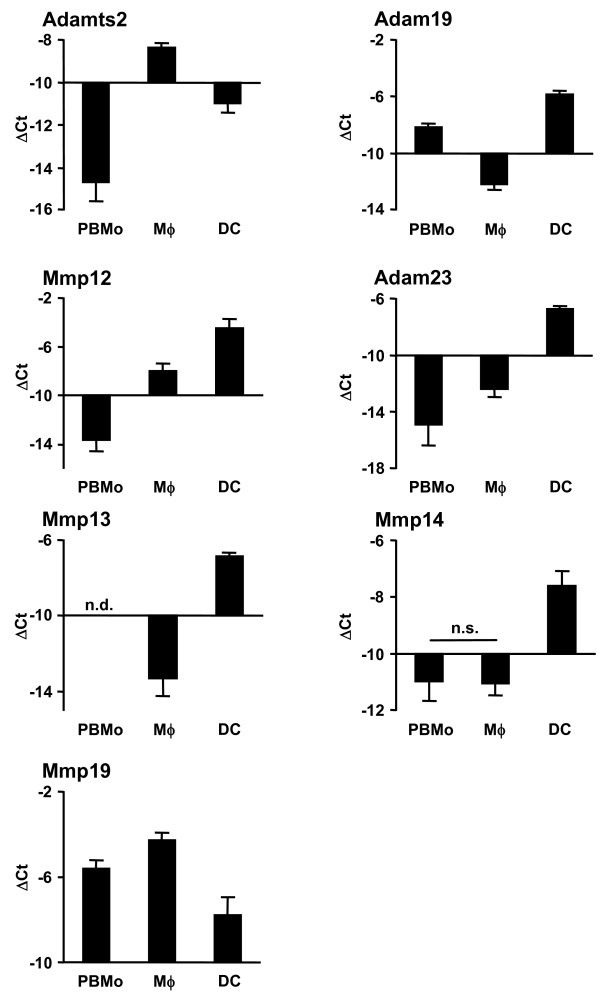
**Validation of metalloproteinase genes by qRT-PCR**. PBMo, lung Mϕ and DC were sorted as shown in Fig. 1A and 2A. mRNA expression was assessed by qRT-PCR analysis for metalloproteinases. Data are presented as mean ± SD of 4 independent experiments per group. All differences between gene expression were statistically significant with p < 0.05 except where indicated by n.s. (not significant). A non-detectable gene expression is indicated by n.d. (not detected).

**Figure 5:**
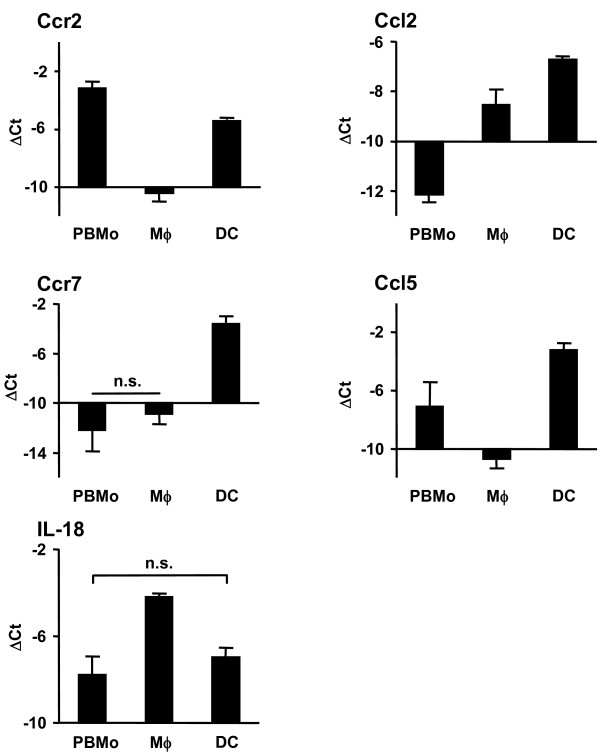
**Validation of chemokine and interleukin genes byqRT-PCR**. PBMo, lung Mϕ and DC were sorted as shown in Fig. 1A and 2A. mRNA expression was assessed by qRT-PCR analysis for chemokines and interleukins. Data are presented as mean ± SD of 4 independent experiments per group. All differences between gene expression were statistically significant with p < 0.05 except where indicated by n.s. (not significant).

**Figure 6:**
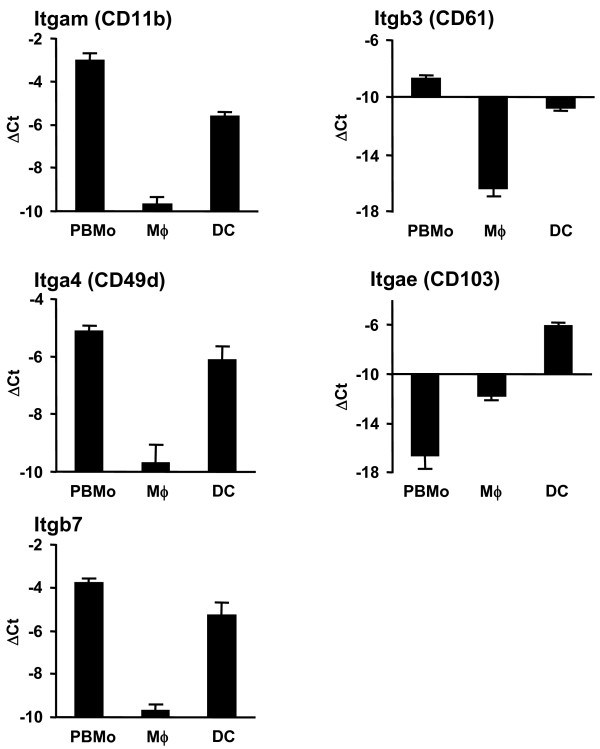
**Validation of integrin genes by qRT-PCR**. PBMo, lung Mϕ and DC were sorted as shown in Fig. 1A and 2A. mRNA expression was assessed by qRT-PCR analysis for integrins. Data are presented as mean ± SD of 4 independent experiments per group. All differences between gene expression were statistically significant with p < 0.05 except where indicated by n.s. (not significant).

To further analyze and structure the microarray data, and to address the question of which gene clusters and cellular pathways are regulated during the extravasations and lung tissue differentiation process of mononuclear phagocytes, particular attention was paid to genes involved in cell trafficking, namely integrins, metallopeptidases, chemokines and chemokine receptors, as well as interleukins and interleukin receptors (Table 1). In order to visualize the results, volcano plots were created with depicted genes belonging to each cluster (Fig. 3). The highlighted genes were validated on independently sorted samples by qRT-PCR and demonstrated the same expression trends as the microarray results (Fig. 4, 5, 6). It must be noted, however, that the log intensity ratios (i.e. the coefficients displayed in Table 1) obtained from the microarray experiments after RNA preamplification do not directly equal the ΔCt values obtained from the qRT-PCR validation. This is a well-known phenomenon and due to partly not well understood factors such as the preamplification procedure itself and the limited dynamic range of fluorescence detection [25,26]. Due to this, ΔCt values obtained from the qRT-PCR analysis were often found to be higher than the coefficients for the same genes obtained from the microarray analysis. Likewise, by qRT-PCR analysis there were significant expression differences detectable in certain genes that had not been detected by the array experiments (Table 1 and Fig. 4, 5, 6).

**Table 1:** Most strongly and significantly regulated genes belonging to selected gene clusters.

**gene symbol**	**gene description**	**coefficient**		
		**MΦ vs PBMo**	**DC vs PBMo**	**MΦ vs DC**
	**metallopeptidases**			

Mmp19	matrix metallopeptidase 19 [NM_021412]	1,99	ND	2,0
Mmp13	matrix metallopeptidase 13 [NM_008607]	ND	3,81	ND
Adam23	disintegrin and metallopeptidase domain 23 [NM_011780]	ND	3,02	ND
Mmp14	matrix metallopeptidase 14 [NM_008608]	ND	2,61	-2,6
Adam8	disintegrin and metallopeptidase domain 8 [NM_007403]	ND	2,50	-3,7
Mmp12	matrix metallopeptidase 12 [NM_008605]	ND	2,39	ND
Mmp8	matrix metallopeptidase 8 [NM_008611]	ND	-2,74	ND
Adam19	disintegrin and metallopeptidase domain 19 [NM_009616]	ND	ND	-2,1
Mmp13	matrix metallopeptidase 13 [NM_008607]	ND	ND	-2,7
Adamts2	disintegrin-like and metallopeptidase	3,65	ND	ND
	with thrombospondin type 1 motif [NM_175643]			

	**chemokine/chemokine receptor**			

Cxcl1	chemokine (C-X-C motif) ligand 1 [NM_008176]	5,69	4,45	ND
Cxcl2	chemokine (C-X-C motif) ligand 2 [NM_009140]	4,76	ND	ND
Cx3cl1	chemokine (C-X3-C motif) ligand 1 [NM_009142]	4,09	4,37	ND
Ccl6	chemokine (C-C motif) ligand 6 [NM_009139]	2,70	ND	2,6
Ccl17	chemokine (C-C motif) ligand 17 [NM_011332]	2,68	4,38	ND
Ccrl2	chemokine (C-C motif) receptor-like 2 [NM_017466]	2,35	ND	ND
Ccl3	chemokine (C-C motif) ligand 3 [NM_011337]	2,25	ND	ND
Cxcl10	chemokine (C-X-C motif) ligand 10 [NM_021274]	2,06	ND	ND
Ccl2	chemokine (C-C motif) ligand 2 [NM_011333]	2,04	2,44	ND
Ccl9	chemokine (C-C motif) ligand 9 [NM_011338]	-2,07	ND	ND
Cxcl4	chemokine (C-X-C motif) ligand 4 [NM_019932]	-2,38	ND	-2,8
Cx3cr1	chemokine (C-X3-C) receptor 1 [NM_009987]	-3,36	ND	-2,7
Ccl5	chemokine (C-C motif) ligand 5 [NM_013653]	-3,72	ND	-5,9
Cxcl7	chemokine (C-X-C motif) ligand 7 [NM_023785]	-4,68	-3,42	ND
Ccr2	chemokine (C-C motif) receptor 2 [NM_009915]	-4,70	-2,04	-2,7
Ccr7	chemokine (C-C motif) receptor 7 [NM_007719]	ND	4,61	-4,4
Cxcl16	chemokine (C-X-C motif) ligand 16 [NM_023158]	ND	4,17	-2,6
Ccl4	chemokine (C-C motif) ligand 4 [NM_013652]	ND	4,09	-3,3
Ccl12	chemokine (C-C motif) ligand 12 [NM_011331]	ND	2,72	ND
Cxcr3	chemokine (C-X-C motif) receptor 3 [NM_009910]	ND	2,55	-4,2
Cxcr4	chemokine (C-X-C motif) receptor 4 [NM_009911]	ND	2,51	-2,6
Ccr9	chemokine (C-C motif) receptor 9 [NM_009913]	ND	2,45	-2,5
Ccl7	chemokine (C-C motif) ligand 7 [NM_013654]	ND	2,26	ND
Cxcr6	chemokine (C-X-C motif) receptor 6 [NM_030712]	ND	ND	-2,6

	**interleukin/interleukin receptor**			

Il1a	interleukin 1 alpha [NM_010554]	4,21	2,25	2,2
Il6	interleukin 6 [NM_031168]	4,18	ND	ND
Il18	interleukin 18 [NM_008360]	3,04	ND	2,6
Il17d	interleukin 17D [NM_145837]	2,69	ND	ND
Il1b	interleukin 1 beta [NM_008361]	2,18	3,84	ND
Il11ra1	interleukin 11 receptor, alpha chain 1 [NM_010549]	2,09	ND	ND
Il2rb	interleukin 2 receptor, beta chain [NM_008368]	-3,14	ND	-5,1
Il12b	interleukin 12b [NM_008352]	ND	3,92	-2,5
Il7r	interleukin 7 receptor [NM_008372]	ND	2,87	-2,7
Il6	interleukin 6 [NM_031168]	ND	2,82	ND
Il18r1	interleukin 18 receptor 1 [NM_008365]	ND	ND	-3,3

	**integrins**			

Itgax	integrin alpha X [NM_021334]	2,39	2,25	ND
Itga2b	integrin alpha 2b [NM_010575]	-2,02	ND	ND
Itgam	integrin alpha M [NM_008401]	-2,11	ND	-2,1
Itga4	integrin alpha 4 [NM_010576]	-2,63	ND	-2,3
Itgb7	integrin beta 7 [NM_013566]	-3,76	ND	-3,5
Itgae	integrin, alpha E, epithelial-associated [NM_008399]	ND	3,88	-3,1
Itgb3	integrin beta 3 [AK135584]	-3,64	ND	-2,8

Genes were selected to keep a false-discovery rate of 10%. Genes are indicated by their consensus name and the NCBI GenBank accession number given in square brackets. The coefficient given for the expression corresponds to log_2 _of fold change with a coefficient >0 indicating upregulation and a coefficient <0 indicating downregulation of the respective gene. Absence of a differential regulation between the respective groups is indicated by ND.

### Isolation and gene expression profiling of subpopulations of PBMo and lung Mϕ

**Figure 7:**
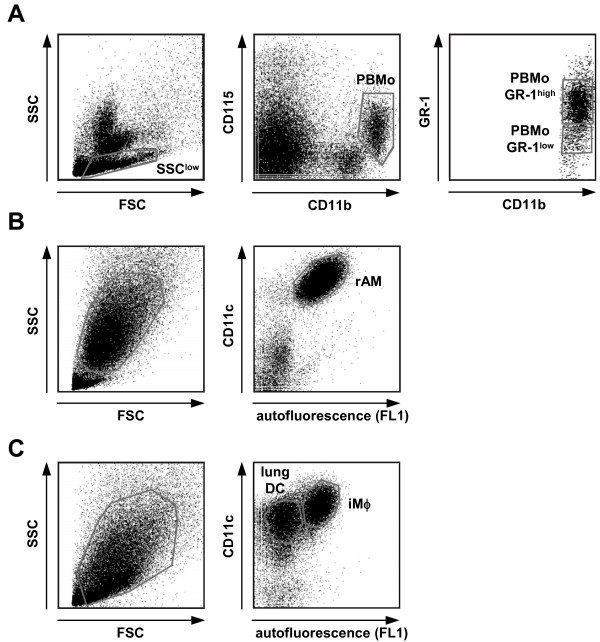
**Isolation strategy of subpopulations of PBMo and lung Mϕ**. **A) **PBMo were flow-sorted from peripheral blood leukocytes by gating on the low SSC/CD11b^pos^/CD115^pos ^population, as shown in Fig. 1. Additional gates were set on the GR-1-positive (PBMo GR-1^high^) and GR-1-negative (PBMo GR-1^low^) subsets of PBMo. **B) **Resident alveolar macrophages (rAM) were flow-sorted from BAL fluid by gating on the high FSC/high SSC/CD11c^pos^/high autofluorescent cell population. **C) **After BAL, lungs were removed, and CD11c^pos ^cells were isolated from lung homogenate using magnetic beads as described. Subsequently, CD11c^pos ^cells from lung homogenate were flow-sorted for the low autofluorescent population representing lung DC and the high autofluorescent population representing Mϕ as described. Note that the majority of rAM had been removed from lungs by lavage prior to homogenization, and that the flow-sorted Mϕ mainly represent interstitial Mϕ (iMϕ). Displayed data are representative of 5–6 independent sorting experiments per group.

The microarray experiments described above were designed to compare the gene expression profiles of PBMo and their fully differentiated pulmonary progeny lung DC and lung Mϕ on a genome-wide scale. This approach, however, does not detect potential differences in gene expression between intermediate differentiation stages or distinct subpopulations of circulating or lung tissue mononuclear phagocytes, which have been ascribed different migratory and differentiation properties. Thus, the two dominant subpopulations of PBMo, the "inflammatory" (GR-1^pos^) and the "resident" (GR-1^neg^) subsets, have been attributed with different biological functions, including recruitment under inflammatory versus steady-state conditions, and differentiation into functionally different DC and Mϕ populations [5,27]. To further identify possible differences in the expression profiles of the selected genes, GR-1^high ^and GR-1^low ^PBMo were sorted for qRT-PCR analysis based on the expression of CD11b, CD115 and GR-1, as depicted in Fig. 7A. Like PBMo, lung Mϕ can be divided into two major populations according to their anatomical location, the parenchymal or interstitial Mϕ (iMϕ), and the resident alveolar macrophages (rAM). Whether these populations represent functionally different subpopulations has long been a matter of debate. Recent reports, however, indicate a functional and developmental difference, with the iMϕ being proposed as precursor cells for rAM [28]. For the separation of rAM, BALF was obtained from mouse lungs, and rAM were flow-sorted from the lavage by gating the high FL1 autofluorescent, CD11c^pos ^cell population (Fig. 7B). By lavaging one can remove > 90% of the alveolar macrophages from mouse lungs [29]. In the current experiments, the lavage procedure depleted rAM efficiently from the lungs thus enriching the iMϕ subset, following an approach used by Landsman et al. [6,28]. No additional rAM could be obtained by serial lavage, indicating an efficient lavaging procedure. Enriched interstitial Mϕ and DC were then isolated from homogenates obtained from lavaged lungs (Fig. 7C) using the sorting strategy described above (Fig. 2A).

**Figure 8:**
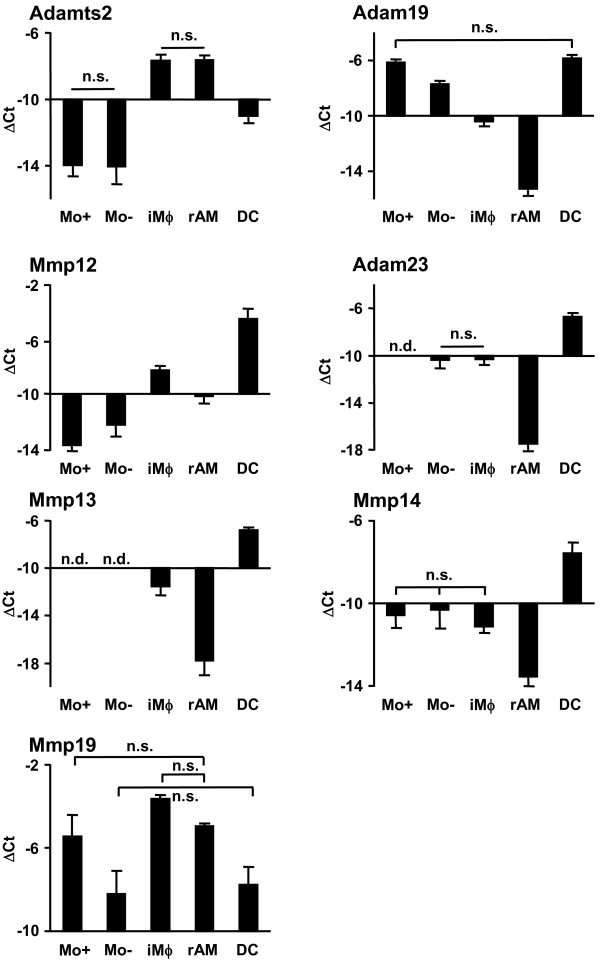
**Relative mRNA expression of metalloproteinase genes by qRT-PCR**. GR-1^high ^and GR-1^low ^PBMo, iMϕ and rAM as well as lung DC were sorted as shown in Fig. 5, and mRNA expression was assessed by qRT-PCR analysis. Data are presented as mean ± SD of 4 independent experiments per group. All differences between gene expression were statistically significant with p < 0.05 except where indicated by n.s. (not significant). A non-detectable gene expression is indicated by n.d. (not detected). Mo-, GR-1^low ^PBMo; Mo+, GR-1^high ^PBMo; iMϕ, interstitial lung macrophage; rAM, resident alveolar macrophage.

**Figure 9:**
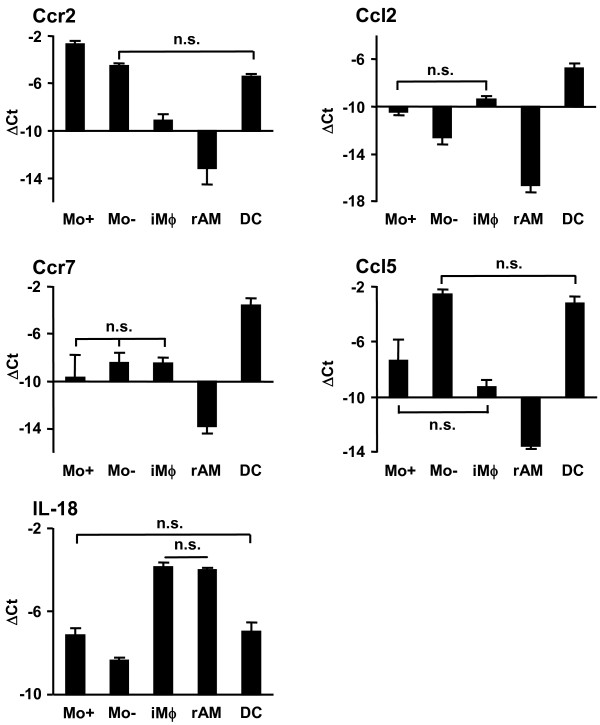
**Relative mRNA expression of chemokine and interleukin genes by qRT-PCR**. GR-1^high ^and GR-1^low ^PBMo, iMϕ and rAM as well as lung DC were sorted as shown in Fig. 5, and mRNA expression was assessed by qRT-PCR analysis. Data are presented as mean ± SD of 4 independent experiments per group. All differences between gene expression were statistically significant with p < 0.05 except where indicated by n.s. (not significant). Mo-, GR-1^low ^PBMo; Mo+, GR-1^high ^PBMo; iMϕ, interstitial lung macrophage; rAM, resident alveolar macrophage.

**Figure 10:**
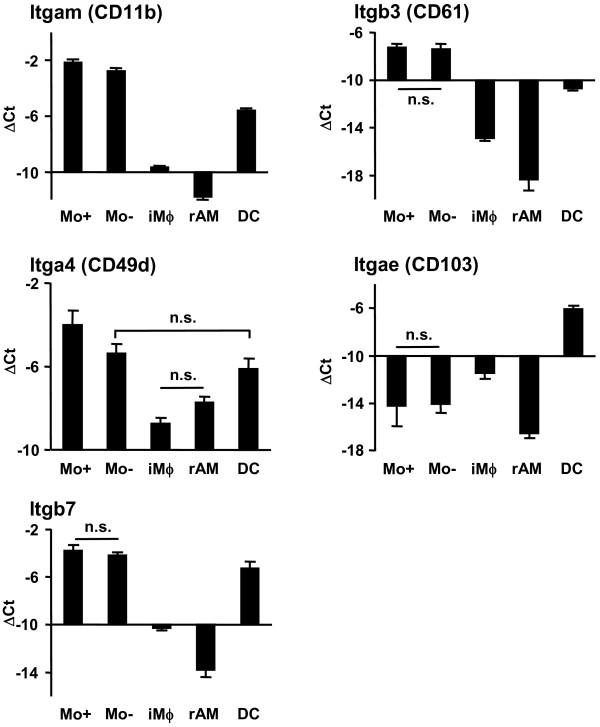
**Relative mRNA expression of integrin genes byqRT-PCR**. GR-1^high ^and GR-1^low ^PBMo, iMϕ and rAM as well as lung DC were sorted as shown in Fig. 5, and mRNA expression was assessed by qRT-PCR analysis. Data are presented as mean ± SD of 4 independent experiments per group. All differences between gene expression were statistically significant with p < 0.05 except where indicated by n.s. (not significant). Mo-, GR-1^low ^PBMo; Mo+, GR-1^high ^PBMo; iMϕ, interstitial lung macrophage; rAM, resident alveolar macrophage.

The differential expression of selected genes was further evaluated in the GR-1^high ^and GR-1^low ^subsets of PBMo, iMϕ and rAM, as well as in lung DC, by qRT-PCR (Fig. 8, 9, 10). Differences in the mRNA expression of all selected genes were statistically significant, and demonstrated the same expression trends as the results obtained by microarray experiments (Fig. 8, 9, 10). In addition to microarray results, new information was obtained with respect to differences in gene expression between subpopulations of PBMo and lung Mϕ. iMϕ and rAM exhibited significantly different gene expression in 14 out of 17 analyzed genes, suggesting a functional and/or developmental difference.

Expression levels of Mmps in PBMo subpopulation were very low or not detectable in qRT-PCR experiments except Mmp14, Mmp19 and Adam19 (Fig. 8). Expression of Mmp19 and Adamts2 did not differ between iMϕ and rAM, but both genes exhibited elevated expression compared to DC. All other Mmps examined exhibited higher expression levels in DC in comparison to iMϕ and rAM.

The GR-1^high ^and GR-1^low ^PBMo subsets did not differ in integrin expression, but significant differences were observed in all other genes analyzed, especially with respect to chemokine and chemokine receptor expression, confirming and expanding previous reports. The expression profile of lung DC was essentially similar to the expression profiles of PBMo subpopulations, while lung Mϕ subpopulations significantly differed.

### Confirmation of selected integrin expression by flow cytometry on subsets of PBMo and lung Mϕ, and lung dendritic cells

**Figure 11:**
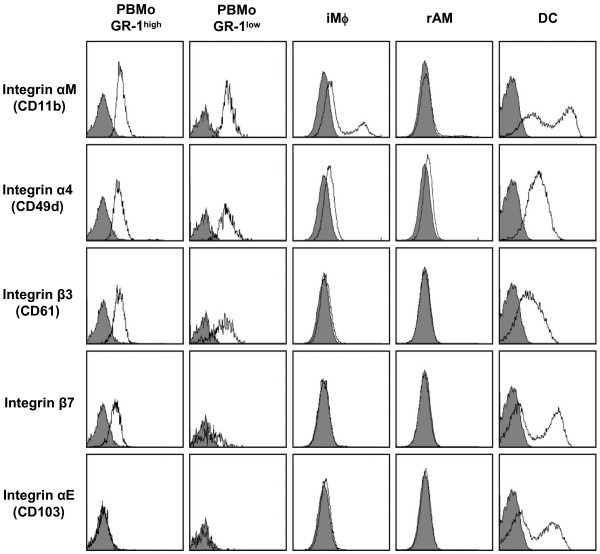
**Confirmation of the expression pattern of differentially regulated integrins with flow cytometry**. GR-1-positive (PBMo GR-1^high^) and GR-1-negative (PBMo GR-1^low^) subsets of PBMo, lung interstitial (iMϕ) and alveolar (rAM) macrophages, and lung dendritic cells (DC) were isolated as described and analyzed by flow cytometry for the expression of the indicated integrins. Gates on the respective cell populations were set as illustrated in Fig. 4. Open histograms indicate specific fluorescence of the indicated antigen; shaded histograms represent control stained cells. Displayed data are representative of three independent experiments.

To further assess whether transcript levels demonstrated the same protein expression pattern, the integrins examined by qRT-PCR on mononuclear phagocyte populations (Fig. 10) were also assessed for cell surface expression by quantitative flow cytometry (Fig. 11). The cell surface expression levels of the respective integrin molecules demonstrated the same expression trends as the observed mRNA levels in the mononuclear phagocyte subsets analyzed. In particular, iMϕ and rAM lose expression of most selected integrins, except integrin α M, which was partially expressed by iMϕ, but was not present in rAM. In contrast, integrin β1, β2 and β3 expression remains high in lung DC. Integrin αE was expressed exclusively on a lung DC subset, and its expression pattern was identical to the expression of integrin β7, suggesting co-expression of integrins αE and β7 on subpopulation of DC, which has previously been described [30].

## Discussion

The constant maintenance of both DC and Mϕ cell pools in the lung is essential for effective immune surveillance in pulmonary tissue. Recent reports highlight the role of PBMo that emigrate into the lung and differentiate into both lung DC and Mϕ, thereby serving as a constant supply for the renewal of the lung DC and Mϕ pool [6]. While many studies have investigated monocyte recruitment under inflammatory conditions, little is known about the pathways mediating monocyte trafficking and differentiation in lung tissue under non-inflammatory conditions [27,31,32]. Since PBMo are believed to be precursors for lung Mϕ and DC, a global gene expression profiling approach was chosen to reveal crucial differences between these cell types, to better understand their relation to one another, and to identify gene clusters relevant for the migration and differentiation process that takes place under steady-state conditions. Previous microarray studies investigating the relation, differentiation and/or maturation of monocytes, macrophages and DC have been mainly conducted *in vitro *using both murine and human cells [33-35]. A study comparing primary human AM versus AM differentiated *in vitro *from PBMo has demonstrated significant differences in gene expression profiles [36], indicating the necessity of carefully elaborating the differences and similarities between *in vivo *and *in vitro *differentiation. A recent publication from our group compared gene expression profiles of murine mononuclear phagocytes recruited to the alveolar space under non-inflammatory and inflammatory conditions using 1 K nylon arrays [37]. The present study was undertaken as the first *in vivo *investigation using cell specific whole genome expression profiling of key players in lung immunity, namely lung Mϕ and lung DC and their circulating precursors PBMo, to define the gene expression differences between these three cell populations under non-inflammatory conditions in mice. By this, it could be demonstrated that approximately 5–10% of all genes are differentially regulated between these three cell populations which are closely related with respect to origin, destination and function. Whether these expression differences represent preformed, lineage-specific differentiation programs or are rather due to the interaction of migrating PBMo and specific micro-environmental factors of the lung must be addressed in detail in subsequent studies.

The gene expression patterns obtained from our study suggest that lung DC are phenotypically closer related to PBMo than lung tissue Mϕ. When further comparing the transcriptional regulation of selected genes in the lung macrophage subpopulations iMϕ and rAM by qRT PCR, the transcripts of CCR2, CCL2, CCR7 and CD61 all highly expressed in PBMo were found to be less downregulated in iMϕ compared to rAM. This finding supports a recent report by Landsman *et al*., suggesting that iMϕ are an intermediate stage in the differentiation process to rAM [28].

An important issue when interpreting DNA microarray data is whether mRNA expression levels demonstrates the same expression trends as the expression of the encoded proteins. Notably, the transcriptional expression patterns of selected integrins obtained by DNA array and qRT-PCR were found to demonstrete the same expression trend as the expression levels of the respective proteins on the cell surface as detected by flow cytometry.

Lung DC and Mϕ play diverse functional roles in innate immunity, and their localization to different compartments of the lung suggests different migration properties of these cell types. Under steady-state conditions, DC largely reside in the interstitial compartment, with only minor parts located in the alveolar space, and ultimately they emigrate to the thoracic lymph nodes to present antigen to T cells. Conversely, Mϕ readily pass through the epithelial barrier and enter the alveolar airspaces, which very likely represents a terminal destination. Hypothesizing that DC and rAM residing in different environments most likely require different migration and tissue invasion capacities, differentially expressed genes were grouped into trafficking related clusters such as integrins, Mmps, chemokine and chemokine receptors, and interleukins and interleukin receptors. Integrins are key mediators of cell-cell interactions, and given their different tissue localization, DC and Mϕ most likely have to interact with different cell types or the extracellular matrix (ECM). Indeed, the paucity of integrin gene expression in rAM as compared to PBMo and lung DC suggests that rAM require less integrin-mediated cell-cell communication, which is consistent with the view of rAM being confined to the alveolar space, rather than possessing extensive migratory properties. Members of the MMP family can cleave components of the ECM, thereby facilitating cell migration [38]. Furthermore, Mmps can modulate the activity of chemokines [39], cytokines [40], and selectins [41]. Chemokines themselves also regulate Mmps and integrin avidity [42-44]. The different biological functions of DC and Mϕ would imply that these cell types have different interactions with their direct environment, suggesting the differential expression of genes that regulate cell interaction with the ECM, and responses to chemokines and cytokines. Our data show that DC and Mϕ express different clusters of MMP as well as cytokine and chemokine receptor genes, indicating distinct patterns of migration properties.

It has previously been shown that Mmp2 and Mmp9 are critically required by DC for recruitment to the airways in a murine model of asthma [45] and for the migration of DC from the skin to lymph nodes [46]. While a difference in gene expression of Mmp2 and Mmp9 between lung DC and lung Mϕ could not be demonstrated by microarray, five members of the Mmp family, Adam19, Adam23, Mmp12, Mmp13 and Mmp14 were identified, which were dramatically upregulated in lung DC versus Mϕ, while Mmp19 was upregulated in both iMϕ and rAM, compared to DC. In contrast, expression of Mmps except Mmp8, Mmp19 and Adam19 in PBMo was low or not detectable, indicating that the transcriptional upregulation of these highly active enzymes is an important and immediate step in the differentiation process after PBMo emigration from the blood into the lung. However, the exact role played by these differentially expressed members of the Mmp family in cell migration, phagocytosis and antigen processing has to be further delineated. Similarly, the mechanisms by which Mmps regulate the function of chemokines, cytokines, and integrin expression, which influence DC and Mϕ migration and activity, also await elucidation. Another important aspect of this study is the detailed delineation of the expression pattern of chemokines and their receptors in PBMo, lung Mϕ and lung DC under non-inflammatory conditions. The microarray and qRT-PCR analyses demonstrate that all three cell populations express a variety of both chemokines and receptors. The qRT-PCR analysis of the mRNA levels in iMϕ and rAM, however, indicates a more active participation in chemokine production by the iMϕ than by the rAM population.

## Conclusion

Taken together, to the best of our knowledge, this study is the first to analyze the gene expression profile of the major phagocytotic and antigen-presenting cells of the lung, Mϕ and DC, and their putative precursor cells, monocytes from peripheral blood, on a whole-genome scale under non-inflammatory, steady-state conditions. The diversity of genes differentially regulated in the investigated clusters was found to be largest in DC corresponding to their volatility and multiple functions in antigen uptake, processing and subsequent presentation. In addition, DC preserve the high expression level of integrin and chemokine/chemokine receptor genes found in PBMo whereas lung Mϕ display much lower transcript levels of these traffic related molecules. As previously poorly investigated players in pulmonary mononuclear phagocyte function, transcript levels of most members of the Mmp family were low or not detectable in PBMo, but were found to be strongly upregulated in both lung DC and Mϕ, however with a unique expression pattern of distinct Mmp family members in both cell types potentially related to cell type specific functions in lung tissue that has to be delineated in further studies.

## Competing interests

The authors declare that they have no competing interests.

## Authors' contributions

ZZ carried out the experimental work and drafted the manuscript. JW did the statistical analysis of the microarray raw data. LC and LMM helped with the qRT-PCR validation of gene expression. WS participated in the experimental design. JL and WW initiated the study, designed the experiments, and participated in the manuscript preparation. All authors read and approved the final version of the manuscript.
